# Cognitive development in children up to age 11 years born after ART—a longitudinal cohort study

**DOI:** 10.1093/humrep/dex102

**Published:** 2017-05-24

**Authors:** Anna Barbuscia, Melinda C. Mills

**Affiliations:** 1 Department of Sociology and Nuffield College, University of Oxford, Manor Road, OxfordOX1 3UQ, UK

**Keywords:** ART, IVF, ICSI, cognitive development, ART children, maternal age, Millennium Cohort Study, confounding factors

## Abstract

**STUDY QUESTION:**

How does the cognitive development of children conceived after ART (IVF and ICSI) – measured as cognitive skills at age 3, 5, 7 and 11 years – differ over time from those born after natural conception (NC)?

**SUMMARY ANSWER:**

Improved measures of cognitive development up to age 5 years were recorded in children conceived with ART compared to NC, which attenuates by 11 years, with ART children still scoring slightly better than NC children.

**WHAT IS KNOWN ALREADY:**

Results on the cognitive outcomes of children conceived after ART have been highly contradictory. Some have shown that ART children have an impaired behavioural, socio-emotional and cognitive development and higher risk of mental disorders. Others have reported no increased risk or difference. Cognitive development has not been previously examined using latent growth curve models from ages 3 to 11 years, also including appropriate attention to confounding parental characteristics.

**STUDY DESIGN, SIZE, DURATION:**

Longitudinal data for the first five waves (2000–2012) of the UK Millennium Cohort Study were used, which is a two-stage sample of all infants born in 2000–2001 and resident in the UK at 9 months of age, drawn from the Department of Social Security Child Benefit Registers. A final sample of *N* = 15 218 children (125 IVF and 61 ICSI), from 14 816 families was used. Information was available for all waves for 8298 children. Four additional follow-up surveys were conducted in 2003, 2005, 2007 and 2012.

**PARTICIPANTS/MATERIALS, SETTING, METHODS:**

Our sample includes children born within a union (married or cohabiting parents) and where information on cognitive scores was available for at least two measurement points. Cognitive development was assessed with the British Ability Scales. At age 3 and 5 years (wave 2 and 3), children completed the naming vocabulary component, which measures expressive verbal ability. At age 7 years (wave 4), verbal cognitive abilities were assessed through the word reading test, and at age 11 years (wave 5) through a verbal similarity test. Two-tailed Student's *t*-tests examined differences between ART and NC groups. Growth curve models (random-coefficient, latent trajectory models) were used to study the effect of ART, confounding parental characteristics and health outcomes at birth, both at a baseline level of cognitive ability at age 3 years and on its growth rate.

**MAIN RESULTS AND THE ROLE OF CHANCE:**

At age 3 and 5 years, children conceived with the aid of ART have higher verbal cognitive abilities than NC children (*P* < 0.001) but this consistently decreases over time and diminishes by age 11 years. Parental environment and resources are pivotal in children's cognitive development.

**LIMITATIONS, REASON FOR CAUTION:**

The sample size of the ART cohort of children is small across each time period (*N* = 150–180) in comparison with NC children (*N* = 10 496–11 955). Owing to a limited sample size, we are also unable to compare IVF versus ICSI treatment.

**WIDER IMPLICATIONS OF THE FINDINGS:**

With the increasing use of IVF and ICSI, these results indicate that there are no detrimental effects on children's early cognitive outcomes up to age 11 years, and highlight the importance of parental characteristics.

**STUDY FUNDING/COMPETING INTEREST(S):**

Funding for this project was provided by the European Union's Seventh Framework Program (FP7 2007–2013) (No. 320116 Families and Societies), ESRC/NCRM SOCGEN Grant (ES/N0011856/1) and the SOCIOGENOME ERC Consolidator Grant (ERC-2013-CoG-615603) (to M.C.M.). The authors have no competing interests to declare.

**TRIAL REGISTRATION NUMBER:**

N/A.

## Introduction

Since the first IVF baby was born in 1978, there has been a rapid increase in ART use, with more than 5 million children conceived to date (Präg and Mills, 2015). This has raised questions regarding the long-term impacts of ART on offspring. Results to date remain highly contradictory. Many studies report an increased risk of an impaired behavioural, socio-emotional and cognitive development (Sutcliffe and Ludwig, 2007; Hart and Norman, 2013a; Spangmose *et al.*, 2017) and mental disorders (Svahn *et al.*, 2015) in ART children. Some studies have found a delay in cognitive development of ART offspring, particularly with ICSI children (Knoester *et al.*, 2008; Goldbeck *et al.*, 2009). ART offspring have higher risks of adverse birth outcomes, such as low birthweight (LBW), preterm delivery and birth defects (van Balen, 1998; Romundstad *et al*., 2008; Wilson *et al.*, 2011; Hansen *et al.*, 2013; Hart and Norman, 2013a; Kondapalli and Perales-Puchalt, 2013; Präg and Mills, 2015). Higher risks of poor health at birth are partially related to the higher incidence of multiple births, a historical consequence of ART (Pison *et al.*, 2015). A series of systematic reviews have concluded, however, that there are no developmental differences between ART offspring and those from a natural conception (NC) once perinatal problems such as preterm birth, LBW and birth defects are taken into account (Wilson *et al.*, 2011; Pandey *et al.*, 2012; Hansen *et al.*, 2013).

Others have likewise found no differences in the cognitive, motor and language development of 2-year-olds (Balayla *et al.*, 2017) and in the intellectual development of 5-year-olds (Ponjaert-Kristoffersen *et al.*, 2004), cognitive outcomes in 3 and 5-year-olds (Carson *et al.*, 2009, 2011), mental health and developmental outcomes at age 7–8 years (Carson *et al*., 2013; Punamaki *et al.*, 2016), overall, verbal and IQ cognitive ability in 8–10 year-olds (Leunens *et al.*, 2006, 2008) or in academic performance of adolescents (Spangmose *et al.*, 2017). Other studies have found not only comparable but also even higher mental health and socio-emotional development of ART children (Wagenaar *et al.*, 2008, 2009; Mains *et al.*, 2010; Hart and Norman, 2013b).

Research to date has generally examined development in different age groups in isolation, rarely scrutinising how differences in cognitive development in ART versus NC children develop over time. Cognitive ability during childhood has been shown to be a pivotal determinant of adolescent and adult outcomes such as educational attainment, earnings, and also crime, participation in risky behaviour, depression and teenage parenthood (Feinstein, 2003; Heckman, 2007). These studies have underlined the importance of looking at progression across different stages of childhood rather than one point in time (Heckman, 2006; Ermisch, 2008; Cunha *et al.*, 2010). Some studies have suggested that infertility and conception via ART may have stronger detrimental impacts in early childhood, related to elevated anxiety, delayed mother–infant attachment, diminished maternal confidence and overprotecting parenting (Bernstein, 1990; Golombok and Brewaeys, 1996; Gibson *et al.*, 2000; Hammarberg *et al.*, 2008). Others have argued that perinatal problems may have long-term negative effects on children's development at older ages, from health conditions to educational attainment and income (Black *et al.*, 2007; Myrskylä *et al.*, 2013).

Recent studies have argued that findings may be confounded by the characteristics of ART parents (Golombok, 2015; Spangmose *et al.*, 2017), yet few have attempted to empirically investigate this selectivity. There is consensus that the early formation of children's cognitive ability is positively related to older, highly educated and high socio-economic status (SES) parents (Feinstein, 2003; Liu *et al.*, 2011; Goisis and Sigle-Rushton, 2014; Myrskylä *et al.*, 2013). Since ART treatments are often costly, access is frequently related to individuals who are able to bear these treatment costs (Chambers *et al.*, 2014a,b; Präg and Mills, 2017).

The aim of this study is to examine whether the cognitive development of children conceived after ART (IVF and ICSI) – measured as cognitive skills at age 3, 5, 7 and 11 years – differs over time compared to children born after NC. A secondary aim is to examine how parental characteristics serve as confounders in these effects.

## Materials and Methods

### Study population

The Millennium Cohort Study (MCS) is a nationally representative prospective cohort study of 18 552 families across the UK. A random two-stage sample of all infants born in 2000–2001 and resident in the UK at 9 months of age was drawn from the Department of Social Security Child Benefit Registers. Ethnically diverse and disadvantaged areas were oversampled to ensure adequate representation. Baseline interviews captured socio-demographic and health information, including questions about pregnancy and fertility treatment. Follow-up surveys were conducted in 2003, 2005, 2007 and 2012. In our sample we only include children born from either married or cohabiting parents, to avoid comparisons with lone mothers (Carson *et al.*, 2009). We consider children who have information available for at least one of the waves after the first measurement, which leads to a final sample of 15 218 children (14 816 families). Information about cognitive scores is available for 11 799 children at wave 2, for 12 125 children at wave 3, for 10 959 children at wave 4 and for 10 643 at wave 5. Analyses were also performed including only children where information is available for all waves (*N* = 8298). The results of the different specifications are virtually identical with the exception that *P*-values are slightly higher in models performed on the balanced sample, also discussed in the Statistical methods section.

### Outcomes and covariates

Children's cognitive development was assessed with the British Ability Scales (BAS II), which is a battery of twelve core sub-tests of cognitive ability and educational achievement, suitable for children aged 2 years and 6 months to 17 years and 11 months, with sound validity and test–retest reliability (Connelly, 2013). We consider standardised BAS II sub-test scores measuring verbal ability. At age 3 and 5 years (wave 2 and 3), children completed the *naming vocabulary* component, which measures expressive verbal ability. At age 7 years (wave 4), verbal cognitive abilities were assessed through the *word reading* test, and at age 11 years (wave 5) through a v*erbal similarity* test, which measure respectively educational knowledge of reading and of expressive verbal ability. The BAS II sub-tests were developed to be age-specific measures of verbal ability and are comparable over time (Connelly, 2013).

To remedy the problem of comparability of test scores across different sets of items, raw scores were converted into standardised *T*-scores and adjusted to take into account the different ages of the children, a particularly important feature since cohort members are born throughout the year and might differ in their ability score for this reason. Standardised scores indicate how a child's cognitive abilities have developed relative to his/her peers (Carson *et al.*, 2011) and were provided as *T*-scores with mean 50 (SD 10). One exception is the *word reading* test at wave 4, for which standardised, but not *T*-scores, were provided. These were computed in an identical manner, but do not have the same mean and SD. To construct the BAS verbal scores and allow us to examine variation over time, we re-standardise the scores based on the mean value and SD in our final sample. The re-standardised scores have all mean = 0 and SD = 1.

The main explanatory variable is whether the child was conceived with the aid of ART, including both IVF and ICSI. In the baseline interview, 186 mothers declared to have conceived after ART (125 IVF and 61 ICSI), making the numbers too small to examine by the type of treatment. Taking into account multiple births, the number of children conceived with the aid of ART is 214. ART offspring are captured with a binary variable (*ART*) taking the value of one if the child was conceived with the aid of one of the two techniques, and 0 otherwise.

Three sets of confounders were included: child's characteristics at birth, parental age and health characteristics and parental SES. Basic demographic characteristics of the child included gender, age and whether she/he was part of a multiple birth (binary variable *multiple*). A first set of explanatory variables concerned the outcomes at birth: whether the child was LBW (*LBW* taking the value of one if the child's weight is <2.5 kg), parity (*first born*) and a variable assessing whether the mother experienced any problems during pregnancy. Unfortunately, the publically available data does not allow us to include precise information on gestational age. We were able to include a measure of very preterm delivery in the growth curve models, which did not alter our results. All information about pregnancy outcomes were reported by the mother in the first wave. The second set included the age of the mother at childbirth and parental socio-economic characteristics, including mother's educational level (*high education* if the mother has a degree or higher educational level) and employment status (*employed*) and a measure of the SES of the head of the household (*high SES*, based on the National Statistics socio-economic classification). Controls for partnership status (*married*), the number of siblings, whether the mother breastfed the child and two measures of economic conditions were also included: whether the household's income is among the UK first quintile, and a measure of deprivation (*OECD *60%, if the household income is lower than the Organisation for Economic Co-operation and Development 60% median). All individual controls are time-invariant factors and the information was provided by parents’ in the first wave.

### Statistical methods

Two-tailed Student's *t*-tests were used to test whether significant differences exist between ART and NC children. In the same way, average BAS cognitive scores of ART and NC children were compared as measured at the different waves. The standardised cognitive sub-test scores provided by the MCS are centred around a mean of 50 and SD of 10, with the exception of wave 4 discussed previously.

Latent growth curve models were used to examine the individual cognitive trajectories of children, which allowed us to study the effect of ART, parental characteristics and health outcomes at birth both on the baseline level of cognitive ability (age) and on its growth rate. These models are special cases of random-coefficient models where the coefficient of time is allowed to randomly vary between subjects and is optimal to examine inter-individual differences in intra-individual change with longitudinal data (Skrondal and Rabe-Hesketh, 2008). Observation can be unbalanced, or in other words, we were able to include children for whom the observation is missing at some time points. Specifications of the same models run on the sample including only balanced observations are available in the Supplementary Table S1; however, the results are virtually identical. Clustered robust SEs were included in the models to account for the high prevalence of multiple births among ART births.

A comparison of model fits between different specifications of the growth curve models (Akaike IC-AIC- and Bayesian IC-BIC-criteria, Supplementary Table S2) and the analysis of variance (Supplementary Table S3) determined that a quadratic random effect model is the most appropriate time metric. Once the optimal baseline growth curve model had been established, predictors were introduced. The effect on both the initial cognitive level and on the growth rate (slope) were examined. The initial cognitive level refers to the first observation, when the children are 3 years old. The main interest lies in the coefficients of ART, showing the effect of ART birth both on the baseline (cognitive ability at age 3 years) and on the growth slope.

A general specification of the model is thus:

(1) $$\begin{array}{c} \mathrm{COG}\mathrm{ij}=\alpha +\beta 1\;*\;{\mathrm{ART}}_{i}+\beta 2\;*\;{\mathrm{ART}}_{i}\;*\;T_{j}+\gamma 1\;*\;T_{j}\\ +\gamma 2\;*\;T_{j}^{2}+\delta 1\;*\;X_{i}+\delta 2\;*\;X_{i}\;*\;T_{j}\\ +\mu_{\mathrm{ij}}+{\mu 2}_{\mathrm{ij}}\;*\;T_{j}+\varepsilon_{i} \end{array}$$

where β1 is the effect of ART on the initial cognitive abilities (at age 3 years), β2 is the effect of ART on the growth rate of cognitive skills, γ1 and γ2 are the linear and quadratic fixed effects of time, and δ1 and δ2 are the random intercept and slope. *X* is a vector including individual controls. Different sets of control variables were included one at a time in the models, including first basic demographic controls, then characteristics at birth and finally parental background characteristics. All predictors included in the models are time-invariant and the characteristics were measured at the first wave. The coefficients predict the random component of the growth trajectories to determine which variables are associated with individuals showing higher or lower intercepts or steeper versus flatter slopes.

## Results

### Health outcomes at birth and parental background

Descriptive statistics (Table I) illustrate consistent differences between children born with and without the aid of ART and most notably a higher prevalence of multiple births among ART births (more than one-third of ART births, compared to the 2% of non-ART births), which was a central consequence of ART in 2000–2001. ART children also have a higher probability of LBW and being born by Caesarean section, with ART mothers more likely to report problems during pregnancy.

**Table I:** Parental characteristics and outcomes at birth, for births following natural conception (NC) and ART.

	NC		ART	
	Mean	SE	Mean	SE
Parental characteristics (family level)				
Mother's age (years)	29.12	0.045	33.86***	0.346
Father's age (years)	30.92	0.069	36.07***	0.496
Married parents (%)	70.59	0.003	92.21***	0.020
Income (first UK quintile, %)	18.87	0.003	37.12***	0.037
Income (below OECD 60%)	27.08	0.003	10.17***	0.023
High SES (managerial or professional occupation of the head of household, %)	30.12	0.003	47.30***	0.038
Mother with degree or equivalent education (%)	38.09	0.003	44.91*	0.038
Mother employed (%)	47.63	0.004	58.68**	0.038
Mother housewife (%)	49.34	0.004	34.73***	0.036
Ever breastfed (%)	70.86	0.003	83.83***	0.028
English first language at home (%)	84.05	0.003	91.10*	0.022
Problems during pregnancy (%)	37.77	0.003	46.10*	0.038
Birth outcomes				
First born (%)	39.47	0.004	75.44***	0.334
Twin (%)	1.20	0.000	22.15***	0.0322
Triplet (%)	0.01	0.000	1.32***	0.013
Number of siblings	0.97	0.008	0.30***	0.046
*N*	14 816		167	
Low birthweight (<2.5 kg, %)	5.25	0.001	19.81***	0.027
*N*	15 004		214	

*Notes*: The stars indicate whether the difference is significant according to a two-tailed* t*-test. Source: Millennium Cohort Study, wave 1, authors’ own calculations. NC, natural conception; OECD, Organisation for Economic Co-operation and Development; SES, socio-economic status. The sample includes only one twin for each multiple birth (except for LBW).

The demographic and socio-economic background of parents who conceived via ART differs markedly from parents with a NC. ART mothers and fathers are on average 4 and 5 years older, respectively. ART parents have a higher income and higher probability of belonging to a high socio-economic class, on average. ART mothers are more likely to have a high educational level and to be employed. All differences are consistent and statistically significant.

### Cognitive development at ages 3–11 years

**Figure 1:**
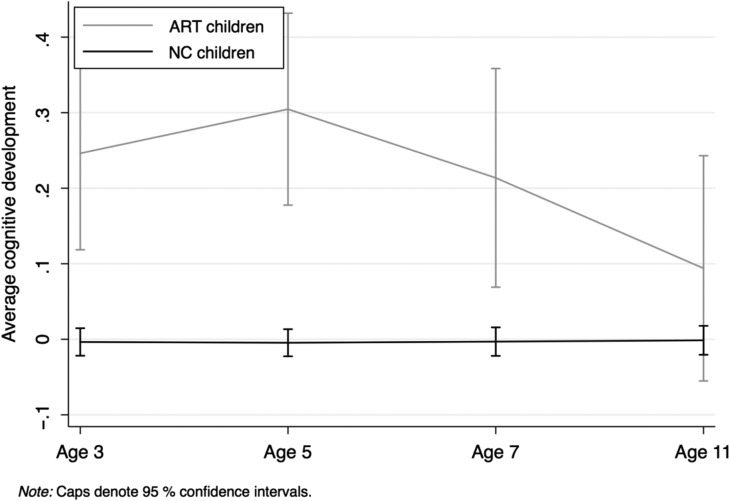
Average cognitive development of children born after ART and natural conception. NC, natural conception. Age in years.

The difference in cognitive scores at different time points (Table II) suggests that, without controlling for any other characteristics, children born with the aid of ART tend to perform better in cognitive tests than NC children. The difference is consistent and statistically significant at waves 2 and 3 (around three points, which is one-third of a SD), but starts decreasing at wave 4, and is not significant at wave 5. This effect is illustrated in Fig. 1, which displays the average cognitive development from age 3 to 11 years of ART versus NC children. A convergent pattern emerges: children born with the aid of ART show significantly higher cognitive levels up to 5 years, but then converging to a very similar level as NC children when they are 11 years old.

**Table II:** Standardised ability scores of British Ability Scale tests measuring verbal ability at each wave, for children born after NC and ART.

BAS Verbal cognitive test score	NC	*N*	ART	*N*	Difference NC versus ART	*P*-value
BAS naming vocabulary (S2)	50.03	11 640	52.88	168	−2.85***	0.0007
BAS naming vocabulary (S3)	54.51	11 955	57.90	180	−3.38***	0.0000
BAS word reading (S4)	112.52	10 802	116.26	157	−3.83**	0.0035
BAS verbal similarity	59.14	10 496	60.08	150	−0.94	0.1234

*P < 0.05; **P < 0.01; ***P < 0.001 according to a two-tailed Student's *t*-test. BAS, British Ability Scale. *Source*: UK Millennium Cohort Study (MCS), wave 2–5, authors’ own calculations.

Table III shows the results of the growth curve models where the dependent variable is the standardised cognitive score of the children and the sets of control variables are included consecutively. Robust clustered SEs account for the high number of twins and triplets among ART births. The coefficients of the variables represent their impact on the initial cognitive level at age 3 years and the coefficients of the interactions between regressors and time represent their impact on the growth rate. In line with what was previously observed (Table II), being born with the aid of ART is significantly associated with higher cognitive skills at age 3 years in the uncontrolled model (coeff. 0.466, *P* < 0.001, Model 5). The association with the growth rate, on the other hand, is not statistically significant, suggesting that birth after ART is linked to early cognitive skills, but not with their development in time. The effect of ART on the cognitive baseline decreases in size but persists when controls for multiple birth, LBW and parity are included (Model 6), suggesting a better performance of ART children despite the poorer health outcomes at birth, which are often negatively linked to cognitive ability. Both the magnitude and the significance of the effect decrease consistently in Model 7, when controls for parental background are added.

**Table III:** Results of growth curve models on BAS Verbal cognitive test standardised scores, waves 2–5 (age 3–11 years).

	Model (5)	Model (6)	Model (7)
ART	0.466*** (0.12)	0.343** (0.12)	0.088 (0.12)
ART*time	−0.0537 (0.030)	−0.0372 (0.031)	−0.0257 (0.031)
Birth outcomes			
Multiple birth	−0.210* (0.11)	−0.0743 (0.11)	−0.139 (0.11)
Multiple birth*time	0.030 (0.027)	0.0104 (0.028)	0.0214 (0.027)
First born		0.397*** (0.030)	0.120** (0.045)
First born*time		−0.0422*** (0.007)	−0.0264* (0.012)
LBW		−0.479*** (0.074)	−0.323*** (0.072)
LBW*time		0.0701*** (0.018)	0.0505*** (0.019)
Parental characteristics			
Mother's age			0.0259*** (0.003)
Mother's age*time			−0.002** (0.032)
Mother's high education			0.262*** (0.041)
Mother's high educ*time			0.002 (0.001)
Employed mother			0.291*** (0.030)
Employed mother*time			−0.043*** (0.008)
High SES			0.165*** (0.036)
High SES*time			−0.0106** (0.0095)
*N*	45 372	45 372	45 372

*P < 0.05; **P < 0.01; ***P < 0.001; robust clustered SE in parentheses. LBW, low birthweight. All models include controls for age and gender of the child. Model (6) includes controls for general problems experienced during pregnancy and whether the pregnancy was planned. Model (7) includes controls about the partnership status of parents, household income, whether the mother breastfed the child, and number of siblings. The sample includes only one twin for each multiple birth (except for LBW).

### Confounding effect of parental characteristics

The specific characteristics associated with ART parents show a strong and positive association with the child's cognitive skills at age 3 years. High education and employment status of the mother have the strongest association with children's cognitive ability together with a high SES of the head of the household. The age of the mother is also positively related to children's cognitive scores, but the effect is considerably smaller. This result provides empirical evidence for previous speculation that there is a positive effect of older, highly educated and high SES mothers on the children's cognitive ability and confirms that the effect is already strong in the first years of life. Interestingly, all parental background factors except for mother's high education tend to have a consistently positive impact on the cognitive baseline level, but no impact or a small negative effect on the rate of growth.

## Discussion

Research has provided very mixed and often contradictory findings regarding whether children conceived with the aid of ART (IVF or ICSI) have lower, similar or better cognitive outcomes in comparison to their NC counterparts. We used a large sample that was representative of the UK population that included children born with and without the aid of ART to examine their cognitive development across five measurement periods from age 3 to 11 years. Our results found a positive association between conception with the aid of ART and children's verbal cognitive abilities as measured by the BAS scale. The effect was strong when cognitive abilities were first measured at the age of 3 and 5 years, but consistently decreased over time and virtually disappeared by age 11 years. The comparability of the measures of verbal cognitive ability over time rules out the possibility that the ‘decline’ in cognitive ability of ART compared to NC children might be related to the different domains assessed by the sub-tests.

These results demonstrate the importance of examining longitudinal data and the trajectory of children's cognitive development across various ages as opposed to taking one ‘snap-shot’ in time, which has been the prominent approach to date. Results suggest that the concerns about a possible negative effect of ART on children's cognitive development are not straightforward and need to be examined in an entire trajectory. Results likewise demonstrate that ART conceived children may even have better cognitive outcomes at very early ages, but also that parental background, and thus the environment and resources that children are exposed to, particularly in the early years of life, is pivotal for development. The positive ‘ART effect’ appears to be mainly attributed to the selective characteristics of ART parents, who are on average older, better educated and have higher SES. This would support the importance of environmental conditions especially for the development of children's early cognitive skills, with differences in cognitive abilities linked to parental characteristics already emerging before the age of 3 years.

The importance of parents’ socio-economic background on the children's development highlights the difference in opportunity experienced by children in the UK. ART children represent a specific subsample and although they are exposed to higher risk of adverse health outcomes at birth, and more likely to be multiple births (Hansen *et al*., 2013; Hart and Norman, 2013a; Pison *et al.*, 2015), which has been linked to poorer cognitive development both in previous studies and in our analyses, the positive effect of parental background effectively ‘overrides’ these negative effects, leading to an overall to a better performance. Our findings echo studies that have found similar positive effects of older maternal age on offspring's outcomes (McLanahan, 2004; Goisis and Sigle-Rushton, 2014). The better cognitive performance of ART children until age of 5 years is in line with previous literature (Carson *et al.*, 2009, 2011) The convergence in the cognitive ability of ART and NC children after 5 years of age and variation in this trajectory, however, is new and takes us beyond previous findings. Until now, previous research demonstrated that differences in cognitive ability emerging at early ages are linked to parental characteristics and largely remain stable over time (Feinstein, 2003; Cunha *et al*., 2010).

The current study likewise suggests that there might be some key mechanisms affecting the cognitive development of ART children that are specifically related to their first years of life. One interpretation is that parents may develop specific ways of parenting and building relationships with these children as they perceive their ART children to be more fragile because of the poorer health outcomes and the long and complex process that led to their birth (Bernstein, 1990; Gibson *et al*., 2000; Carson *et al*., 2011). Such perceptions could lead to particularly attentive ways of parenting when the child is very young, which might change as the child grows up healthy and, for example, starts going to school. Once the child is not perceived as particularly fragile, parents might develop alternative ways of parenting that are simply in line with their SES, leading to a smaller difference in the performance of the two groups of children. The strong need and considerable psychological and financial efforts to have an ART child undoubtedly also contribute to the differences and higher attention in parenting.

The study suffers from some important limitations. First, the sample size of the cohort of ART children is not ideal and consistently smaller than that of NC children. Furthermore, it does not allow us to distinguish between the effects of different kinds of ART, which has been highlighted in previous studies (Ponjaert-Kristoffersen *et al.*, 2004; Knoester *et al.*, 2008; Goldbeck *et al.*, 2009). Another limitation is that we did not directly explore the role of parenting in the link between parental background and ART and children's development, which would need a dedicated separate study to be fully addressed.

Nevertheless, the results of this study provide an important contribution to the existing knowledge about the outcomes of ART and add a unique contribution to the literature exploring the impact of ART and early life conditions on the cognitive development of children. Information about cognitive skills at different points in time and the rich information about parental background permitted us to explore the effect of different factors on the development of ART children over time as opposed to single snap-shots. The dynamic approach is particularly important as the effects of birth after ART are likely to differ depending on the age of the child. The inclusion of twins and triplets in our analyses adds to the previous literature, which usually excluded multiple births or examined them separately from singletons. Considering the high prevalence of multiple births across ART births, excluding twins and triplets can lead to partial results and a consistent loss of information.

The stronger performance of ART children despite the higher prevalence of adverse early health outcomes would support the idea that children growing up in certain SES conditions and with parents that are committed and have the resources to have children overrides possible adverse health outcomes at birth.

## Supplementary data

Supplementary data are available at *Human Reproduction* online.

## Supplementary Material

Supplementary Data

Supplementary Data

Supplementary Data
