# Supplementary material for: Cognitive development in children up to age 11 years born after ART—a longitudinal cohort study
Source: Hum Reprod. 2017 May 24;32(7):1482–8. doi: 10.1093/humrep/dex102 (PMC5850752; doi:10.1093/humrep/dex102)
Supplement: Supplementary Data [file dex102_suppl_table1.pdf]

Human Reproduction, 2017
doi:10.1093/humrep/dex102

human reproduction

SUPPLEMENTARY DATA

Supplementary Table SI Results of growth curve models on British Ability Scale Verbal cognitive test standardised scores, waves 2–5 (age 3–11 years), balanced sample.

	Model (5)	Model (6)	Model (7)
ART	0.386** (0.13)	0.193* (0.12)	0.058 (0.13)
ART*time	−0.0600 (0.031)	−0.0447 (0.30)	−0.0328 (0.031)
Birth outcomes			
Multiple birth	−0.258 (0.13)	−0.116 (0.13)	−0.183 (0.13)
Multiple birth*time	0.059 (0.0310)	0.0331 (0.031)	0.044 (0.031)
First born		0.418*** (0.035)	0.0141* (0.054)
First born*time		−0.0446*** (0.008)	−0.0276* (0.014)
LBW		−0.527*** (0.088)	−0.396*** (0.082)
LBW*time		0.0921*** (0.021)	0.0715*** (0.021)
Parental characteristics			
Mother's age			0.024*** (0.0039)
Mother's age*time			−0.002*** (0.001)
Mother's high education			0.166*** (0.039)
Mother's high educ*time			0.013 (0.010)
Employed mother			0.185*** (0.035)
Employed mother*time			−0.046*** (0.009)
High SES			0.135*** (0.041)
High SES*time			−0.005 (0.010)
N	33 192	33 192	33 192

*$P < 0.05$; **$P < 0.01$; ***$P < 0.001$, SE in parentheses.
Balanced sample refers to only the cases where we had information available for individuals at all time points. All models include controls for age and gender of the child. Model (6) includes controls for general problems experienced during pregnancy and whether the pregnancy was planned. Model (7) includes controls about the partnership status of parents, household income, whether the mother breastfed the child, number of siblings.
LBW, low birthweight; SES, socio-economic status.
