# Supplementary material for: Cognitive development in children up to age 11 years born after ART—a longitudinal cohort study
Source: Hum Reprod. 2017 May 24;32(7):1482–8. doi: 10.1093/humrep/dex102 (PMC5850752; doi:10.1093/humrep/dex102)
Supplement: Supplementary Data [file dex102_suppl_table2.pdf]

Supplementary Table SII **Model fit comparison.**

Model	Observations	LL	AIC	BIC
(1) Empty	44 182	−173 077.6	346 161.2	346 187.2
(2) Linear	44 182	−171 908.6	343 827.2	343 870.7
(3) Quadratic fixed, linear random time effects	44 182	−171 699.8	343 411.7	343 463.9
(5) Quadratic fixed, quadratic random time effects	44 182	−171 699.8	343 405.7	343 431.8

Notes: Model (1) is the random intercept-only model; Model (2) includes a linear random slope for the time; Model (3) includes a fixed quadratic effect for time; Model (4) includes a random quadratic effect for time.
Akaike (AIC) and Bayesian (BIC) Information Criteria are measures of 'deviance' statistics: smaller values are to be preferred.
AIC = −2LL + 2(*n* parameters)
BIC = −2LL + log(*N*)(*n* parameters)
LL is the natural logarithm of the likelihood function.
