# Supplementary material for: Cognitive development in children up to age 11 years born after ART—a longitudinal cohort study
Source: Hum Reprod. 2017 May 24;32(7):1482–8. doi: 10.1093/humrep/dex102 (PMC5850752; doi:10.1093/humrep/dex102)
Supplement: Supplementary Data [file dex102_suppl_table3.pdf]

Supplementary Table SIII **Growth curve models on BAS verbal ability scores, wave 2–5.**

	Model (1)	Model (2)	Model (3)	Model (4)	Model (5)	Model (6)	Model (7)
var(cons)	38.5972	40.37487	41.0148	41.0148	40.86232	38.67529	27.03729
var(time)		2.57E–13	2.81E–13	1.07E–17	9.29E–17	7.88E–16	5.91E–12
var(time2)				1.64E–20	1.45E–19	9.83E–21	5.83E–14
var(residuals)	119.0699	110.8817	109.3013	109.3013	108.9374	109.1357	108.0581

Model (1): Empty model.
Model (2): Linear random intercept model.
Model (3): Quadratic fixed linear random intercept model.
Model (4): Quadratic random intercept model.
Model (5): ART and basic demographic predictors model.
Model (6): ART, demographic and outcomes at birth predictors.
Model (7): ART, demographic, outcomes at birth and parental background predictors.
